# Menopause and the healthcare workforce: a scoping review and stakeholder consultation

**DOI:** 10.1186/s12913-025-13906-z

**Published:** 2026-01-29

**Authors:** Jessica Scott, Jason Hancock, Morwenna Rogers, Jacqui McBurnie, Karen Mattick

**Affiliations:** 1 Department of Health & Community Sciences, Faculty of Health and Life Sciences, University of Exeter, Exeter, UK; 2 Devon Partnership Trust, Exeter, UK; 3 Northeast and North Cumbria Integrated Care Board, Sunderland, UK; 4 NHS England, Tonbridge, United Kingdom

**Keywords:** Menopause, Perimenopause, Healthcare, Workplace, Workforce, Interventions, Support

## Abstract

**Background:**

Menopause is a transition experienced by all women that is considered a taboo subject in most workplaces globally. Menopause leads many women in healthcare to reduce their working hours, or to leave work, which means that patients and learners do not have access to their expertise. There is limited research exploring how healthcare staff experience menopause and the impact of the loss of senior women on junior staff. This study aimed to understand how the problem of the menopause at work in healthcare settings is characterised in the literature and to explore the implications of menopause at work.

**Methods:**

A scoping review was conducted using Arksey and O’Malley’s framework, including the 6^th^ optional stage of stakeholder consultation. The searches were run on databases MEDLINE, EMBASE, PsycINFO, Social Policy and Practice and HMIC via OVID and CINAHL, via EBSCOhost combining terms for health personnel, workplace and menopause. We conducted thorough supplementary searches in addition to the database searches.

**Results:**

Seventy-five articles from 13 different countries were included, of which 39 were empirical research. These revealed a vicious cycle: experiencing the menopause at work makes work more stressful and, when work is stressful, the experience of the menopause is exacerbated. Support interventions were widely discussed but there was no evidence of the interventions being tested for effectiveness. There were three main themes of implications for women experiencing menopause: implications for perception of self, implications for relationships at work and implications for work/career. There was minimal discussion about implications for colleagues and patients and none about implications for learners.

**Conclusions:**

We have highlighted a small body of empirical research on the topic of the menopause in the healthcare workplace and the implications of this. Further research is required to understand the effectiveness of the interventions being used and to understand the implications for health professions education.

**Supplementary information:**

The online version contains supplementary material available at 10.1186/s12913-025-13906-z.

## Background

Menopause is a transition experienced by all women that is considered a taboo subject in the workplace globally [1, 2]. The average age of menopause is 51 and there are 657 million women worldwide aged 45–59 years. Forty seven percent of these women are employed and a proportion of these will be experiencing physical and psychological symptoms associated with the menopause that is impacting their ability to work. It is estimated that by 2030 the world population of menopausal and post-menopausal women will increase to 1.2 billion [3].

Menopause is preventing women from working. One third of women report taking time off due to menopause symptoms but only one quarter of these women informed their employer that menopause was the reason [4], thus the true impact of menopause is underestimated. Multiple organisations worldwide have highlighted the importance of considering the menopause and adapting working environments for women, to support women to stay in work [2, 5, 6], but data suggests [7] that currently women still aren’t getting the support they need to facilitate this.

The implication for the healthcare workforce is clear. Three quarters of the UKs National Health Service (NHS) workforce is female [8] and 19% of the total NHS workforce was of menopausal age in 2019 [9]. The financial impact of menopause in nurses, health-visitors and midwives alone in England has been estimated at £166 million annually [9]. The NHS has committed to supporting women working through the menopause with the creation of the guidance “Supporting our NHS people through menopause: guidance for line managers and colleagues” [10].

In the UK 10% of women felt unable to work when experiencing menopause [7] with many more reducing hours or changing role [1]. A further group of women working in healthcare settings continue to work despite feeling unwell (so called ‘presenteeism’). These themes are repeated in reports produced by professional organisations. The British Medical Association (a trade union and professional body for UK doctors) reports that 90% of the doctors with menopausal symptoms felt that the menopause impacted their working lives [1]. The Medical Protection Society (a mutual protection organisation for medical, dental and healthcare professionals) report highlights that 20% of doctors either experiencing or having experienced menopause considered retiring early [11].

The potential implications for patients are clear. However, there are wider implications, for example for colleagues and learners in healthcare environments, who need access to motivated and experienced staff for supervision and role modelling. Absenteeism, presenteeism and early retirement of experienced staff creates a gap in the healthcare workforce that particularly affects learners. Early career workers are supported by senior role models, clinical supervisors and mentors. If they are exposed to fewer female healthcare workers as leaders they may come to believe it is not possible for women to have lifelong careers, achieve progression or undertake senior roles.

There is a limited but growing literature exploring how healthcare workers experience symptoms of menopause at work and the impact it has on them, patients, and colleagues, and now it needs to be scoped and synthesised. Healthcare organisations are recognising the importance of the menopause but there is a limited understanding of how staff experience symptoms, the implications of it and if career approaches are helpful. Understanding the nature of the problem and the implications of experiencing the menopause for healthcare workers is a pre-requisite for any further research.

We will be discussing menopause from a bio-psycho-social perspective, focusing on healthcare workers individual experiences in the workplace, with patients and their colleagues, rather than a medical model that is focused on hormonal changes and medical treatment.

## Methods

The aim of the study was to understand how the problem of the menopause at work in healthcare settings is characterised in the literature and to explore the implications of menopause at work.

### Study design

The research team worked within a constructivist paradigm taking the position that knowledge is created through interaction. A scoping review methodology as described by Arksey and O’Malley [12] was chosen as an appropriate methodology within this paradigm. The study was conducted as per the 6-stage framework.

### Stage 1: identifying a research question

This review was interested in the experiences of women who are healthcare workers during the perimenopause or menopause. We were interested in their own experiences of the peri/menopause, not how they support patients with menopause. The context of this review was any healthcare workplace in all countries globally. We developed the questions from initial reading of the literature and discussions within the research group and the advisory group.

The research questions were:

1. What has been published about how menopause is experienced at work by healthcare workers?
2. What support interventions in healthcare work settings have been discussed in the literature?
3. What is known about the implications of menopause experiences at work for healthcare workers themselves or others (e.g. colleagues, learners, patients)?

Definitions for terms used can be found in Additional File 1.

### Stage 2: identifying relevant articles

To develop a comprehensive search strategy, initial familiarisation of articles on the topic was used to identify key words. A pilot search strategy was developed with MR, information specialist, and tested using databases and citation chasing to identify key papers and search terms. Full searches were run on the databases MEDLINE, EMBASE, PsycINFO, Social Policy and Practice and HMIC via OVID and CINAHL, via EBSCOhost. We combined terms for health personnel, workplace and menopause. We used Medical Subject Heading terms (MeSH), which are official words or phrases selected to represent particular biomedical concepts. Terms are organised into a hierarchy called the MeSH tree. We used a combination of free-text terms (in the title and abstract fields) and MeSH terms. Searches used in this study used exploded MeSH terms meaning that all subtypes within the tree were included. For example the MeSH term Health Personnel includes over 30 terms for healthcare roles (full details of the health personnel MeSH tree, with all of the terms included in the search are found in Additional file 2). The full search was developed on MEDLINE and adapted for other databases and is available in Additional File 2. We conducted supplementary searches, backwards and forwards citation chasing, to capture any records not found by the database search strategy. We then inputted the search terms “healthcare personnel” and “menopause” into google and screened the first 100 results.

### Stage 3: study selection

All the articles found were exported into Rayyan (a freely available online systematic review platform) which was used by the research team for screening.

Inclusion and exclusion criteria were developed after initial familiarisation of the literature (Table 1).

**Table 1:** Inclusion and exclusion criteria used in the scoping review

Included	Excluded
• Any form of healthcare worker • Healthcare workers are the focus of the experience • The experience is the menopause • Any healthcare workplace • Any country • Any language	• Healthcare workers treating/supporting patients through menopause • Focus not on healthcare workers experience • Vets • Conference abstracts

Articles from the google search had to meet the same inclusion and exclusion criteria as the articles sourced from the data-bases search. They could be web-articles, reviews, or documents as this scoping review considered a wide range of articles, published by organisational bodies, third sector organisations or media outlets. This was because we anticipated a relatively small number of empirical research papers and some rich accounts in other article types.

Researcher JS independently reviewed all the titles and abstracts with the second screening split equally between JH, KM and MR.

Those articles meeting the inclusion criteria on title and abstract review, or where there was any uncertainty of categorisation, were assessed at the full text level. JS read all and JH, KM and MR divided the articles so that each article was read by two reviewers independently. Any disagreements at the full text review stage were discussed at a whole team meeting to decide the papers to be included.

Any articles written in a language other than English were translated using the Google Lens translate function. The pages of the documents were photographed allowing the Google Lens translate software to superimpose the translation onto the document allowing it to be read.

### Stage 4: charting the data

The data from all articles was charted into a data extraction form in Excel by JS. This form included the healthcare profession and setting, information relating to the experience of menopause and support interventions. The articles were read line by line by JS and the findings were reviewed at regular team meetings to check and discuss the data extraction.

### Stage 5: data analysis and presentation

The charted data from all included articles were then collated to answer research questions 1 and 2 with tables providing an overview of the published literature and interventions experienced/sought by health workers.

A subset of 9 included articles was then analysed to answer research question 3, using narrative analysis [13]. The articles were selected by the research team as having sufficient contextual richness to enable an understanding of the implications at work. Articles were read line-by-line. Text relating to the implications of the menopause was highlighted. For this question we were led by the data, using an inductive approach without a pre-established coding frame [13]. Thematic categories were iteratively identified and discussed at regular whole team meetings.

### Stage 6: consultation exercise

An advisory group was convened at the start of the project to support it throughout and ensure the outputs were relevant and useful. It consisted of experts in menopause and its consequences on health (DB and CD), and a researcher with prior experience in evaluating the menopause in the workplace (AM). They gave feedback throughout the study on the data-analysis and interpretation of the results. While discussing interpretations of the results with the advisory group a conceptual model was developed to illustrate the relationship between how the menopause is experienced and its implications. Multiple iterations of the model were discussed, drawing on the included literature and the team and advisory group’s experience of the literature and healthcare/educational practice.

### Team reflexivity

Constructivist research accepts that personal and professional experiences of the research team will shape the research process and findings. Our team comprised of clinicians (JS, JH and JM), including one who supports healthcare workers experiencing the menopause (JM), an information specialist (MR) and a professor of medical education (KM). Throughout the research process, in regular team meetings, we discussed how these experiences might impact the research.

## Results

### What has been published about how menopause is experienced at work by healthcare workers?

**Fig. 1:**
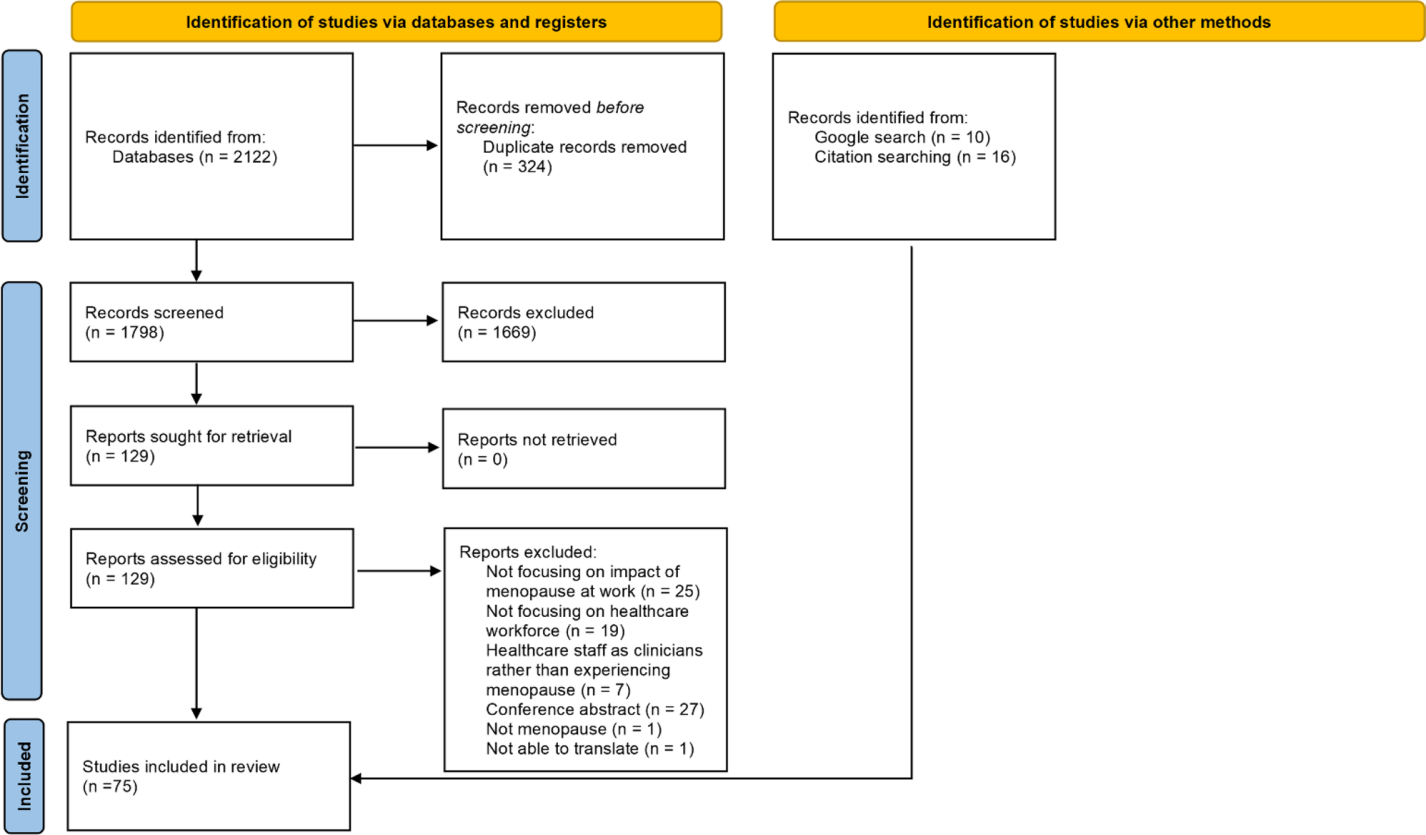
PRISMA flow diagram

We included 75 articles in total (Fig. 1), published between 1995 and 2024, of which 39 were empirical research studies and 36 were non-empirical. The 75 articles were from 13 different countries, including the UK (43), Brazil (8) and China (4). A range of professions were researched or discussed, including nurses (31), mixed healthcare workers (26), doctors (9) and dentists (4). The empirical studies included qualitative (14), cross sectional analysis (19), cohort studies (2), mixed methods (3), and a literature review (1). More details of all included articles can be found in Additional File 3.

### What support interventions in healthcare work settings have been discussed in the literature?

Support interventions were discussed in 65% of the included articles overall (86% of non-empirical articles, and 46% of empirical articles).

Table 2 outlines the range of interventions discussed, which included existing interventions and recommendations or suggestions for interventions from participants or authors. The majority of articles discussed multiple interventions. Four national guidance documents/policies that encompassed many of the interventions were highlighted in the papers: the Royal College of Midwives’ Working With The Menopause document [53], the Royal College of Nursing’s Menopause Guidance [54], the NHS Employers’ Menopause Guidance [10], and the NHS Scotland’s Menopause Policy [55].

**Table 2:** Interventions discussed in the articles with description and citations

Intervention	Description	Citated by
Workplace policy/guideline	A document includes specific guidance on how line managers can support their staff experiencing the menopause.	[1, 9, 14–37]
Menopause training/education/awareness	Sessions for employees who support others (often mangers) going through the menopause developed to give information that may include education about menopause and the implications.	[11, 14–17, 19, 22, 25, 27–29, 32–35, 38–45]
Flexible working	Allowing employees to adapt their hours, location, or how they work to meet their needs or mitigate their symptoms.	[1, 9, 11, 15, 18, 26, 28, 31, 32, 35–37, 39, 42, 44–47]
Changes to working environment	Examples include ensuring water is available, changing the temperature, allowing fans.	[1, 15, 17, 19, 22, 31–33, 39, 42, 46]
Reasonable adjustments	A change made to an employee’s working conditions or arrangements to compensate or adjust for an aspect of that person’s disability.	[1, 9, 18, 22, 25, 36, 37, 41, 43–45, 48–50]
Menopause conferences/workshops/seminars	Spaces for employees to come together to learn about menopause and specific issues in the workplace.	[14, 16, 20, 25, 31, 46, 51, 52]
Changes to uniform	Uniform changes to be more breathable and comfortable.	[16, 26, 32, 33, 35–37, 42, 44]
Peer support	A system to connect employees experiencing the menopause with others who are either going through it or have been. This support is offered in the workplace on an informal, peer support basis.	[16, 41, 45, 51]
Support for mental health and wellbeing	Specific services to support the mental health and wellbeing for employees.	[1, 17, 37]
Risk assessments	The development of a risk assessment with an employees manager to review the risks of continuing to work in the current situation and identify any workplace changes that could be made.	[31–33]
Staff menopause clinic	Clinic available at place of work for employees to have appointments with a clinician. Offers range of treatments.	[26, 52]
Menopause champions	An individual within an organization who takes on a dedicated, proactive role in raising awareness, providing support, and driving positive change related to menopause in that organization.	[9, 18, 42]
Training from Henpicked	Training provided by Henpicked by menopause in the workplace experts. They provide training, videos, eLearning and policy and communications expertise for the organisation.	[34, 42]
Personal protective equipment advice	Royal college of nursing recommending reduction in time PPE is worn.	[30, 33]
Occupational health support	Support from the organisation’s occupational health team.	[11, 45]
Menopause café	Events for people to gather for a coffee, eat cake and discuss menopause.	[51]
Menopause project group	A group developed to support women during the menopausal transition. The aims of the group were to develop a variety of materials and resources designed to assist staff going through or support those going through the menopause transition.	[20]
Communication strategy	Regular briefings and updates within the monthly Chief Nurse bulletins issued to enable and improve communications around menopause between managers and their staff, and to circulate positive organisational messages about the subject.	[20]

There were no examples of interventions being tested for effectiveness.

### What is known about the implications of menopause experiences at work for healthcare workers themselves or others (e.g. colleagues, learners, patients)?

Thematic analysis of nine qualitative, contextually rich studies [14–18, 38, 51, 56, 57] identified three main themes of implications for women experiencing menopause: implications for perception of self, implications for relationships at work, and implications for work/career (Table 3).

**Table 3:** The three themes and subthemes from thematic analysis, the implications of the menopause for women experiencing it

Themes: Implications for those experiencing menopause	Subthemes	Quotes
1. Implications for perception of self		*“I feel like an idiot. It’s like a fog, I can’t process anything”* [19] *“I am known for performing at 110%, but now feel that I am performing below par - which affects my self-esteem and makes me anxious about the security of my job (even while being aware that is not a real risk)”* [22]
2. Implications for relationships at work	Relationships with patients Relationships with colleagues	*“you have to look down into a wound and you have to remove some beads [of sweat] as it drips from your forehead”* [19] *“It is just crap. Sometimes seeing the patients … I’m having to desperately scan through pages and pages of notes to remember who they are”* [51] *“If I was to take time off work because of my symptoms I would probably be laughed at by management”* [21] *“On a really bad day - I get to the point where inside I am a moment away from losing it and wiping the floor with anyone who looks the wrong way or says the wrong thing (yes my rage can be this extreme!)”* [22]
3. Implications for work/career	Trying to cope with work Making changes to job Time off due to sickness Not progressing in role Leaving/retiring early	*“Because of my age, menopausal symptoms and poor sleep I find it extremely difficult to do night shift and get home safely and sleep well. I have already ran off the road and crashed my car on the way home from night shift but no one cares. I still have to do my share of night shifts”* [57] *“and I actually reduced my hours because … I just couldn’t cope with life really anymore and I couldn’t cope with a full-time job*” [51] *“I had to phone in sick whilst trying to get a doctor to review my dosage. I explained to my supervisor exactly why I was off sick but on returning my back to work form did not state this as the reason!*” [16] *“I now have no ambition to progress any further, really … .I’m really enthusiastic about my new role. But I don’t ever want to get to a point where I was so stressed and unwell before.”* [18] *“I see male colleagues of same origin as male supervisor get training which improves promotion and I have more experience and am left “to rot”: other male colleagues just ignore me even when I’m placed in an overseeing role but the top manager”* [16] *“There have been many times I have thought of resigning my job because of the impact of my peri-menopause symptoms.”* [15] *“I resigned from the NHS 5 years ago as couldn’t cope with my life and body going through the menopause since the age of 45 symptoms got worse and more problematic.”* [16]

### Theme 1: implications for their perception of self

Women in healthcare roles reported that around the time of the menopause they perceived themselves to be not as good at their job as they previously were. Reduced levels of confidence were often reported which was linked with reduced memory and concentration as well as high anxiety.

*I am known for performing at 110%, but now feel that I am performing below par - which affects my self-esteem and makes me anxious about the security of my job (even while being aware that is not a real risk)* [16]

### Theme 2: implications for relationships at work

Participants reported that the menopause impacted on two types of relationships. Those with patients and those with colleagues.

Women in healthcare roles reported a change in interactions with patients due to them experiencing symptoms of menopause. For example, hot sweats led to embarrassment in front of patients, due to high levels of sweating or the need to change clothes. Memory difficulties have been highlighted as causing women to not remember things about patients that they would have done previously.

*It is just crap. Sometimes seeing the patients … I’m having to desperately scan through pages and pages of notes to remember who they are* [51]

Relationships with colleagues appeared to change around this time, driven by the perception that they did not understand the implications of the menopause for the women experiencing it. The idea of the symptoms of menopause not being recognised or taken seriously by their peers or managers may lead to women not seeking support they need.

*If I was to take time off work because of my symptoms I would probably be laughed at by management*^*21*^

### Theme 3: implications for their work/career

The implications for women’s work and career was widely reported. The need to cope was very prevalent, and this could take different forms with some women feeling as though there was no option but to attend work even if they felt too unwell to do so.

Some women reported adjustments to working patterns with a reduction in hours or demotion feeling like the only option. The feeling of needing to take a demotion to cope appears to be closely linked to the feeling that some women have of not being able to progress any further.

*I now have no ambition to progress any further, really … .I’m really enthusiastic about my new role. But I don’t ever want to get to a point where I was so stressed and unwell before* [18]

Not all women felt able to make adjustments. Taking time off sick was described, although some women did not feel able to report menopause as the reason.

Reducing hours and taking sickness absence has impacts on the workforce but they are only temporary. The option of leaving the workforce was discussed a lot amongst the women interviewed, and while some were contemplating this, others made the decision to leave.

*I resigned from the NHS 5 years ago as couldn’t cope with my life and body going through the menopause since the age of 45 symptoms got worse and more problematic.* [16]

Staff expressed concern for the impact of taking off work on their colleagues in two of the nine papers [16, 18].

*As nursing staff, we don’t have the ability often to take time away to gather ourselves and we put a lot of pressure on ourselves if we have to take sick leave as we know we are leaving our colleagues short staffed.* [16]

There was not sufficient information to answer the question when focusing on implications for learners.

A small number of studies [17, 38] acknowledged that the patient experience could be negatively impacted if health workers were themselves being impacted by the menopause.

## Discussion

The aim of this scoping review was to understand how the problem of the menopause at work in healthcare settings is characterised in the literature and to explore the implications of menopause at work. We found that there is a body of empirical research published on the topic, describing the problem. However, we found no research testing the efficacy of the vast number of interventions that were described. 48% of the articles found in this scoping review were non-empirical and these, as well as the empirical studies mainly contained self-reports of experiences and difficulties in the workplace. Objective data such as the financial cost or days of work lost due to menopause is not widely reported and when it is, it is an estimate due to limits in the methods [9] or poor use of menopause as a sickness code [58]. We have also found that there is a lack of research on the experiences of healthcare workers that are not healthcare professionals. There were no articles focusing solely on healthcare workers that are not healthcare professionals and only 34% of articles looked at the experiences of a mixture of healthcare workers, which did include experiences of administrative staff.

**Fig. 2:**
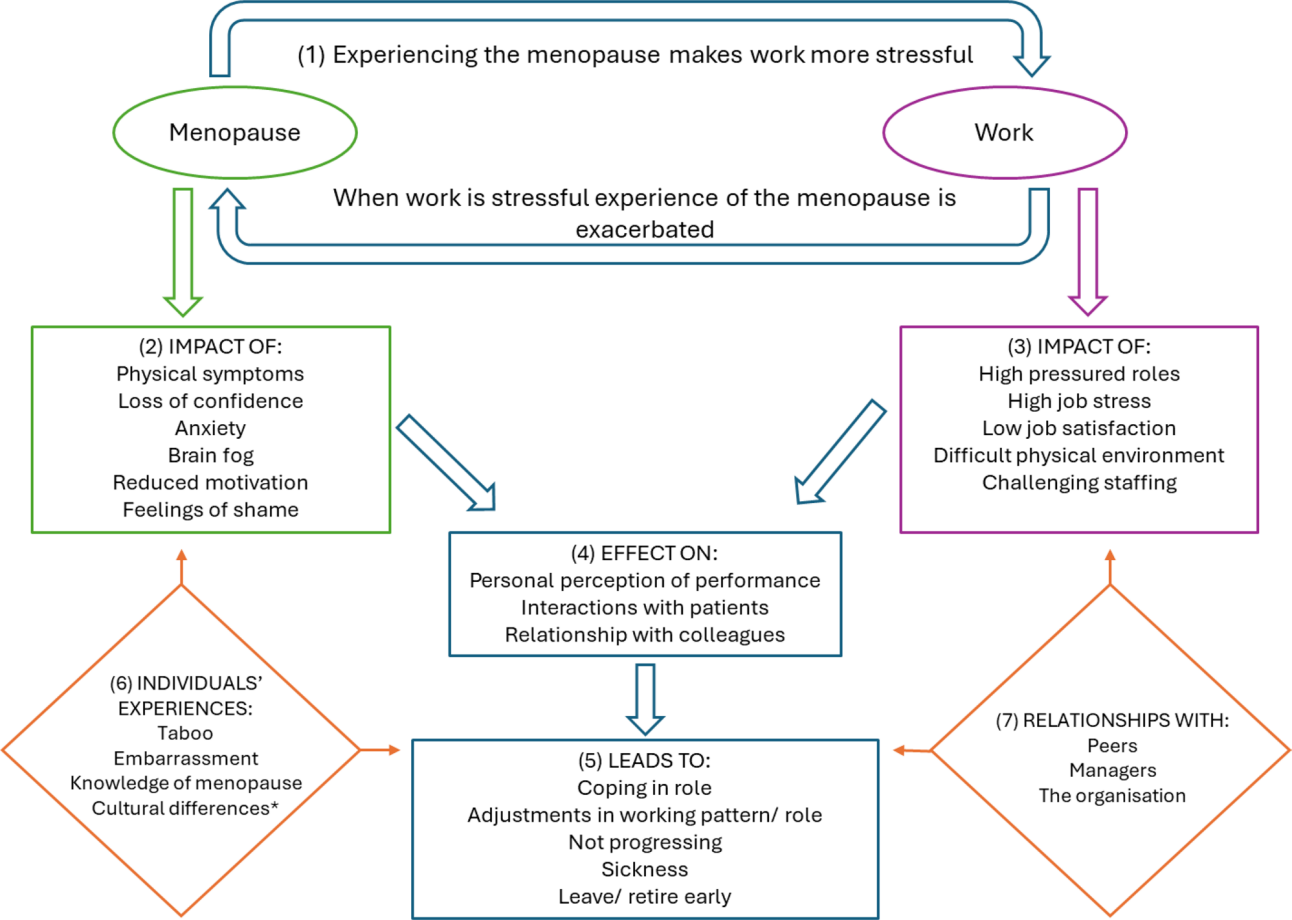
Conceptual model. (Use the numbers in brackets to help move around the model) Experiencing the menopause makes work more stressful and in return when work is stressful the experience of the menopause is exacerbated (1). Specific symptoms of menopause(2) have been highlighted as being problematic at work, and alongside specific issues in the workplace (3) healthcare workers identify that there is an effect on how they interact with patient, how they perceive their performance and ability at work and how they interact with their colleagues. These appear to be the main drivers of the outcome (5) which is different for each individual. There seem to be at least two factors which can impact or mitigate the effects of menopause and work and change the outcome. How an individual experiences the menopause (6) can change how they experience the symptoms of menopause and the decision they make to stay or leave work. In addition, the relationships that a healthcare worker has at work (7) can affect the outcome, positively or negatively. *Cultural differences in how individuals experience menopause was not discussed widely in the research but was significant within the advisory group discussion

Working with our advisory group we propose a conceptual model to support understanding of the interactions between work and menopause (Fig. 2). It has been developed based on our interpretation of the included literature and team discussions. Our hope is that as further research is conducted into this important field others can continue to modify or adapt this model to their own context.

The conceptual model illustrates a vicious cycle: experiencing the menopause makes work more stressful and when work is stressful the experience of the menopause is exacerbated [14, 39, 40, 51, 59–63]. Specific symptoms of menopause that are discussed as being problematic at work [15, 18, 46, 56, 64–67] can be seen to be contributing, alongside the specific workplace issues experienced [15, 16, 40, 66, 68]. Both influence how the women perceive their own performance [17, 18], including patient interactions and colleague relationships [69, 70].

This vicious cycle leads to significant implications for the women experiencing the menopause such as leaving their profession [18, 19, 39], as they feel unable to fulfil their role to the level that they previously did.

It also explores how an individual experiences the symptoms of menopause [38, 56, 61] and the relationships they have at work [38, 47, 51, 66, 70], appear to be two factors that can impact or mitigate the effects of menopause at work.

The implications of the menopause on the women experiencing it are widely discussed as being negative for the individual and the health service. Whilst it is reassuring that the importance of supporting healthcare workers is recognised, the fact that no empirical research studies have tested the effectiveness of such interventions is concerning. Dennis and Hobson [71], researching all workplaces, concluded that the evidence base was lacking around how staff experiencing the menopause are best supported at work. Therefore, it is perhaps not surprising that our study of the healthcare workforce demonstrated a lack of evidence.

## Implications for research, practice and health professions education

Healthcare workplaces are places of learning. For many healthcare workers, supporting students, trainees or those early in their career is a routine and important part of their role. In addition, healthcare professionals have a professional obligation to engage in continuous learning and professional development throughout their career. Therefore, if there are implications of the menopause in the workplace, then there will be implications for learners. If one’s senior role model or mentor is experiencing negative effects at work due to the menopause, whether that is coping, making adjustments or leaving the profession, it is easy to see that this will be impacting on those they are supporting. Understanding what these implications are for those experiencing the menopause, and those that they support, will be an important part of future work.

For women experiencing the menopause, the implications for their own learning and development is briefly discussed in the literature. Women describe not feeling able to progress with their career either due to their own perception of themselves, or others not supporting their ongoing learning. The individual’s experiences of taboo, embarrassment, knowledge of the menopause and their cultural background, as well as the relationships they have with their peers, managers and the organisation are important factors in why women either stay and cope, make adjustments, stop progressing, or leave the profession. These would be areas of interest when planning future research to understand which interventions are effective and why.

The interventions that have been developed and are being used in the workplace may not be effective and could even be causing harm, so evaluating them is important for future research. Wellbeing is a complex system-level problem and interventions typically address different aspects of this problem. Therefore, it may be helpful to use existing frameworks [72] to ensure implicit beliefs about how an intervention might work are mirrored in the experiences of the people receiving them. This work will need to involve the health workers themselves as well as the people designing the interventions. Understanding what works, for who and why is a good place to start when thinking about what is currently being used and what needs to change to better support the workforce.

## Strengths and limitations

The paper describes a rigorous scoping review conducted by a multidisciplinary team with a motivated and engaged advisory group who contributed to multiple parts of the study design and interpretation. The results of the study can be used globally as a wide range of articles based in different locations and published in different languages were used.

As with all research studies, there are some limitations. This study focuses on female healthcare workers and there is little discussion about other people who may experience the menopause, including people assigned female at birth but not identifying as a woman. The results should therefore be considered in this context and further research will be needed to better understand the different experiences of trans men and non-binary people.

We have focused on the problem of the menopause in this study and we have found that for a lot of women it is a problem. However, we have not fully explored the voices of the women who would describe that it has no implications for their work. We acknowledge that the conceptual model developed is only one way of interpreting the experiences and implications of the menopause in the workplace.

The quality of the articles included were not assessed, in line with scoping review methodology [12], but a high proportion of results were non-empirical. We translated articles using GoogleLens so that we were able to include all articles identified. It is likely to produce a less nuanced translation compared to using a translator and may lead to a reduced rigour in our interpretation of the themes from these articles. All the results were used in the study so not to miss important views from opinion and commentary pieces which are often produced by the people experiencing a problem. However, we acknowledge the potential limitations of this approach.

## Conclusion

The aim of this scoping review was to understand how the problem of the menopause at work in healthcare settings is characterised in the literature and to explore the implications of menopause at work. We have highlighted a small body of empirical research on the topic of the menopause in the healthcare workplace and the implications for women and others around them and identified a gap in the research focusing on interventions. There is a clear interaction between menopause and the workplace and understanding how interventions could be used to reduce the negative nature of the interaction is an important next step. Further research is required to understand the effectiveness of the interventions being used already and to understand the implications for health professions education.

## Electronic supplementary material

Below is the link to the electronic supplementary material.

Supplementary Material 1

Supplementary Material 2

Supplementary Material 3

## Data Availability

All data generated or analysed during this study are included in this published article and is available on request.

## References

[1] BMA. Challenging the culture on menopause for working doctors [Internet]. 2022. [cited 2023 Feb 6]. Available from: https://www.bma.org.uk/advice-and-support/equality-and-diversity-guidance/gender-equality-in-medicine/challenging-the-culture-on-menopause-for-working-doctors-report.

[2] Rees M, Bitzer J, Cano A, Ceausu I, Chedraui P, Durmusoglu F, et al. Global consensus recommendations on menopause in the workplace: a European menopause and andropause Society (EMAS) position statement. Maturitas. 2021, Sep;151:55–62. 34274202 10.1016/j.maturitas.2021.06.006

[3] Hill K. The demography of menopause. Maturitas. 1996, Mar;23(2):113–27. 8735350 10.1016/0378-5122(95)00968-x

[4] CIPD. Press release: majority of working women experiencing the menopause say it has a negative impact on them at work. [Internet]. 2019. [cited 2022 Dec 29]. Available from: https://www.cipd.co.uk/about/media/press/menopause-at-work#gref.

[5] Department of Health and Social Care. Women’s health strategy for England policy paper, updated august 2022. Cp. 2022;736. [Internet]. [cited 2022 Dec 29]. Available from: https://www.gov.uk/government/publications/womens-health-strategy-for-england/womens-health-strategy-for-england#menopause.

[6] Royal College Obstetrics and Gynaecology. Improving the health and wellbeing of girls and women better for women [Internet]. 2019. cited 2023 Feb 6]. Available from: https://www.rcog.org.uk/media/h3smwohw/better-for-women-full-report.pdf.

[7] Bazeley A, Marren C, Shepherd A. Menopause and the workplace [Internet]. 2022. [cited 2023 Feb 13]. Available from: https://www.fawcettsociety.org.uk/menopauseandtheworkplace.

[8] Lindsay J, May R. NHS Blog: NHS England signs menopause workplace pledge. [Internet]. 2022. [cited 2022 Dec 29]. Available from: https://www.england.nhs.uk/blog/nhs-england-signs-menopause-workplace-pledge/.

[9] Mulla A, Wiltshire J, Lucas S, Duggal S, Jones E, Moore E, et al. Menopause and the NHS workforce [Internet]. The midlands decision support network. 2022. [cited 2023 Jul 17]. Available from: https://www.strategyunitwm.nhs.uk/sites/default/files/2022-11/Menopause%20and%20the%20NHS%20Workforce%20-%20%20NHS%20Strategy%20Unit%20Report%20.pdf.

[10] NHS England. Supporting our NHS people through menopause: guidance for line managers and colleagues [Internet]. 2022. [cited 2023 Feb 6]. Available from: https://www.england.nhs.uk/wp-content/uploads/2022/11/B1329-guidance-Supporting-NHS-people-through-menopause-November-2022.pdf.

[11] Medical Protection Society. Supporting doctors through the menopause policy paper [Internet]. 2022. [cited 2023 Feb 6]. Available from: https://mpscdnuks.azureedge.net/resources/docs/mp/consultatation-responses/2208314266-menopause-campaign-policy-paper.pdf.

[12] Arksey H, O’Malley L. Scoping studies: towards a methodological framework. Int J Soc Res Methodol. 2005, Feb;8(1):19–32.

[13] Braun V, Clarke V. Using thematic analysis in psychology. Qual Res Psychol. 2006, Jan;3(2):77–101.

[14] Cronin C, Abbott J, Asiamah N, Smyth S. Menopause at work-an organisation-based case study. Nurs Open. 2024, Jan, 1;11(1). 10.1002/nop2.2058 PMC10721947 38268277

[15] Prothero LS, Foster T, Winterson D. ‘Menopause affects us all …’: menopause transition experiences of female ambulance staff from a UK ambulance service. Br Paramed J. 2021, Dec, 2;6(3):41–48. 34970081 10.29045/14784726.2021.12.6.3.41 PMC8669643

[16] Riach K, Lee M, Mavromati K. Advancing menopause and menstrual health in organisations (AMMInO): a National study of employees in health and Social Care. Internet. 2023. Glasgow Available from: https://publichealthscotland.scot/publications/scottish-health-service-costs/scottish-health-service-costs-high-level-costs-summary-2021-to-2022/].

[17] Vanderzalm J, Deschenes S, Kunyk D. Women’s health nurses’ experiences of menopause. Nurs Manage [Internet]. 2023, Jun;54(6):34–40. Available from: https://journals.lww.com/10.1097/nmg.0000000000000023. 10.1097/nmg.0000000000000023 PMC10226387 37194925

[18] Ali S, Duggal S, Hextell L, Jones E, Mobeen M, Moore E, et al. Menopause and the NHS workforce qualitative report [Internet]. 2023. [cited 2024 Sep 25]. Available from: https://www.strategyunitwm.nhs.uk/sites/default/files/2023-10/Menopause%20and%20the%20NHS%20workforce%20-%20QUALITATIVE%20Report%202023.pdf.

[19] Pitman S, Adams N. Menopause in the workplace and you. World Of Ir Nurs And Midwifery. 2022, Mar;30(2):22–24.

[20] Banks S. Menopause and the NHS: caring for and retaining the older workforce. Br J Nurs. 2019;28(16):1086–90. 31518539 10.12968/bjon.2019.28.16.1086

[21] Barker A. Managing menopause. Dent Nurs. 2024;20(1):31–35.

[22] Bell JA, Garlick D, Stevens C. It’s time to talk about the M word. Br Dent J. Springer Nat. 2022;232:15–17. 10.1038/s41415-021-3817-y PMC8758981 35031734

[23] Calow A, Morrell-Scott N, Johnson Smith E. An overview of menopause, and why this should feature within pre-registration education. Br J Nurs. 2023;32(7):334–40. 37027417 10.12968/bjon.2023.32.7.334

[24] Critchley J, Schwarz M, Baruah R. The female medical workforce. Vol. 76. Anaesthesia. Blackwell Publishing Ltd; 2021. p. 14–23. 10.1111/anae.15359 33682097

[25] Davies M, Clyburn P, Barker P, Flatt N, Noble N, Swart M, et al. Age and the anaesthetist: considerations for the individual anaesthetist and workforce planning. Anaesthesia. 2022, Nov, 1;77(11):1259–67. 36173018 10.1111/anae.15825

[26] Dean E. Managing hot flushes and making friends with PPE. Nurs Standard. 2020;35(11):8–10.

[27] Anon. Menopause in the workplace [Internet]. Frontline. 2024. [cited 2024 Jul 22]. Available from: https://www.csp.org.uk/frontline/article/workplace-wins-20.

[28] Hamoda H, Morris E, Marshall M, Kasliwal A, de BA, Bms RM, Rcog RCGP, FSRH. FOM and FPH position statement in response to the BMA report ‘challenging the culture on menopause for doctors’. Vol. 27. Post Reproductive Health. SAGE Publications Ltd; 2020; 2021 August. p. 123–25. 10.1177/2053369120985319 33499740

[29] Kitney V. Being a menopause friendly employer [Internet]. DDU. 2023. [cited 2024 Jul 22]. Available from: https://www.theddu.com/guidance-and-advice/latest-updates-and-advice/being-a-menopause-friendly-employer.

[30] Kydd A. Menopause and personal protective equipment: how does this meet acceptable working conditions? In: Case reports in Women’s health. Vol. 32. Elsevier B.V; 2021. 10.1016/j.crwh.2021.e00356 PMC8441062 34540597

[31] Noble N. Helping and supporting staff to manage menopause symptoms at work. Nurs Times. 2021;117(6):39–41.

[32] Norton W, Tremayne P. How nurse leaders can support staff going through the menopause. Vol. 27. Nursing Management. RCN Publishing Company Ltd; 2020. p. 22–27. 10.7748/nm.2019.e1893 31886637

[33] Anon. Support for menopause at work. Nurs Manage. 2021;28(6):16–17.

[34] O’hearn S. Place-based experiences in the work environment during the menopausal transition: a Case study of Canadian physiotherapists. 2023.

[35] Thomas S. Nurse going through the menopause? Advice from a healthcare professional who’s been there [Internet]. Balance. 2022. [cited 2024 Jul 22]. Available from: https://www.balance-menopause.com/menopause-library/nurse-going-through-the-menopause-advice-from-a-fellow-healthcare-professional-whos-been-there/#:~:text=Talk%2520to%2520colleagues%2520and%2520your,give%2520vital%2520avenues%2520of%2520support.

[36] Dean E. Calling time on a culture of silence. Nurs Standard. 2019;34(11):35–38.

[37] Dean E. Menopause: why nurses need help from their employers [Internet]. Nursing standard. 2018. [cited 2024 Jul 22]. Available from: https://rcni.com/nursing-standard/features/menopause-why-nurses-need-help-their-employers-132511.

[38] Cronin C, Bidwell G, Carey J, Donevant S, Hughes KA, Kaunonen M, et al. Exploring digital interventions to facilitate coping and discomfort for nurses experiencing the menopause in the workplace: an international qualitative study. J Adv Nurs. 2023, Oct, 1;79(10):3760–75. 37700454 10.1111/jan.15679

[39] O’neill MT, Jones V, Reid A. Impact of menopausal symptoms on work and careers: a cross-sectional study. Occup Med (chic Ill). 2023, Aug, 1;73(6):332–38. 10.1093/occmed/kqad078 PMC10540666 37542726

[40] Na PY, Chen CX, Liu GY. Analysis of impact factor of menopausal symptoms of clinical nurses in Tangshan city. Zhongguo Fuyou Baojian. 2014;29(3):309–92.

[41] Devlin R. Who’s helping nurses through menopause? Nurs Standard. 2019;34(11):39–40.

[42] Holland C. Taking the taboo out of the menopause - how dentistry is grappling with “the change. BDJ Team. 2022, May, 20;9(5):12–14.

[43] O’Sullivan SA. Pause for thought: the impact of the menopause on women in the workplace. J Plast Reconstr Aesthet Surg. Churchill Livingstone. 2023;87:369–70. 10.1016/j.bjps.2023.10.083 37939641

[44] Waters A. NHS must offer flexible working to staff with menopausal symptoms, says guidance. BMJ. 2022, Nov, 24;o 2845. 10.1136/bmj.o2845 36423923

[45] Webber A. NHS menopause guidance recommends flexible working [Internet]. Personnel Today. 2022. [cited 2024 Jul 22]. Available from: https://www.personneltoday.com/hr/nhs-menopause-guidance.

[46] Hickey M, Riach K, Kachouie R, Jack G. No sweat: managing menopausal symptoms at work. J Psychosom Obstet Gynaecol. 2017, Jul, 3;38(3):202–09. 10.1080/0167482X.2017.1327520 28532219

[47] Converso D, Viotti S, Sottimano I, Loera B, Molinengo G, Guidetti G. The relationship between menopausal symptoms and burnout. A cross-sectional study among nurses. BMC Womens Health. 2019, Nov, 27;19(1). 10.1186/s12905-019-0847-6 PMC6882317 31775724

[48] Cornock M. Menopause: how reasonable adjustments could help. Nurs Manage. 2022;29(6):10–11.

[49] Cornock M. Menopause symptoms: the adjustments at work that could help. Nurs Standard. 2022;37(11):43.

[50] Anon. Get workplace support during menopause. Nurs Standard. 2022;37(7):6.

[51] Hobson G, Dennis N. “I can’t be dealing with this brain fog”: a workplace focus group study investigating factors underpinning the menopausal experience for NHS staff. Maturitas. 2024, Feb, 1;180. 10.1016/j.maturitas.2023.107889 38029510

[52] Noble N. Making the menopause more manageable. Occup Health Wellbeing. 2019;13–15.

[53] Royal College of Midwives. Working with the menopause [Internet]. 2022. [cited 2024 Sep 25]. Available from: https://rcm.org.uk/publications/working-with-the-menopause/.

[54] Royal College of Nursing. Menopause: RCN guidance for nurses, midwives and health visitors. second edition [Internet]. 2020. [cited 2024 Sep 25]. Available from: https://www.rcn.org.uk/Professional-Development/publications/rcn-menopause-guidance-for-nurses-midwives-and-health-visitors-uk-pub-009326.

[55] Scotland NHS. INTERIM national menopause and menstrual health policy for NHS SCOTLAND [Internet]. 2023. [cited 2024 Sep 25]. Available from: https://wellbeinghub.scot/resource/mmhp/.

[56] Cavalcante SMBS, Catrib AMFC, da Silva RM, Frota MA. O CLIMATÉRIO E SUA RELAÇÃO COM A SAÚDE E O AMBIENTE DE TRABALHO. RBPS. 2006;19(3):140–47.

[57] Riach K, Jack G. Women’s health in/and work: menopause as an intersectional experience. Int J Environ Res Public Health. 2021, Oct, 1;18(20). 10.3390/ijerph182010793 PMC8536086 34682537

[58] Mulla A, Wiltshire J, Lucas S, Duggal S, Jones E, Moore E, et al. Menopause and the NHS workforce quantitative analysis [Internet]. 2023. [cited 2024 Sep 25]. Available from: https://www.strategyunitwm.nhs.uk/sites/default/files/2023-10/Menopause%2520and%2520the%2520NHS%2520workforce%2520-%2520QUANTITATIVE%2520Report%25202023_0.pdf.

[59] Bapayeva G, Terzic M, Semenova Y, Sarria-Santamera A, Gusmanov A, Aimagambetova G, et al. Unveiling the role of the work environment in the Quality of life of menopausal physicians and nurses. Int J Environ Res Public Health. 2023, Sep, 1;20(18). 10.3390/ijerph20186744 PMC10531365 37754604

[60] Cao F, Hoa Y, Lei P. Logistic regression analysis on influencing factors of perimenopause in clinical nurses. Hebei Med J. 2014;15:2355–57.

[61] Ding L, Xie L, Mao J, Zhou Q. Factors influencing perimenopausal syndrome in clinical nurses: a qualitative study. Ann Palliat Med. 2022, Jan, 1;11(1):146–54. 35144406 10.21037/apm-21-3572

[62] Lei N. Effects of work environment and social support on perimenopausal syndrome among clinical nurses in Tianjin. Continuing Med Educ. 2015, Aug;29(8):79–81.

[63] Matsuzaki K, Uemura H, Yasui T. Associations of menopausal symptoms with job-related stress factors in nurses in Japan. Maturitas. 2014;79(1):77–85. 25082207 10.1016/j.maturitas.2014.06.007

[64] Beneventi MCT, Lima SMRR. Morbidities and medications used by practicing nurses during the climacteric. Rev Assoc Med Bras. 2021;67(11):1706–11. 34909902 10.1590/1806-9282.20210773

[65] Reis L M, Loiola Moura A, Carmo Lourenço Haddad M D, Terezinha Oliveira Vannuchi M, Nogueira Smanioto F. Proceso Trabajo De E DE. Influência do climatério no processo de trabalho de profissionais u in a public univ hosp influencia del climaterio en. Abr. 2011;16(2):232–41.

[66] Fonsêca TC, Giron MN, Berardinelli LMM, Penna LG. Quality of life on climacteric nursing professionals. Revista da Rede de Enfermagem do Nordeste. 2014, Jun, 16;15(2).

[67] Giron MN, Campos Fonseca T, Marcia L, Berardinelli M, Helena L, Penna G. Repercussions of the climateric among nurses-an exploratory study. Online Braz J Nurs [Internet]. 2012, Dec;11(3):736–50. Available from: http://www.objnursing.uff.br/index.php/nursing/article/view/3862http://www.objnursing.uff.br/index.php/nursing/article/view/3862.

[68] Stock D, Knight JA, Raboud J, Cotterchio M, Strohmaier S, Willett W, et al. Rotating night shift work and menopausal age. Hum Reproduction. 2019, Mar, 1;34(3):539–48. 10.1093/humrep/dey390 PMC7210710 30753548

[69] Benetti IC, Sales LDS, Deon APDR, Wilhelm FA, Junior JPR. Climatério, enfrentamento e repercussões no contexto de trabalho: vozes do Extremo Norte do Brasil. Rev Kairos. 2019, Mar, 30;22(1):123–46.

[70] de F RAV, Aam D, Dos Santos RM, Paiva Alves ER. CLIMATÉRIO: perspectivas de mulheres profissionais da saÚde do hospital universitÁrio nova esperanÇa, joÃo pessoa-pb. Revista de Ciências da Saúde Nova Esperança. 2020, Aug, 31;18(2):61–72.

[71] Dennis N, Hobson G. Working well: mitigating the impact of menopause in the workplace - a narrative evidence review. Maturitas. 2023, Nov;177:107824. 37634294 10.1016/j.maturitas.2023.107824

[72] Pearson A, Carrieri D, Melvin A, Bramwell C, Scott J, Hancock J, et al. Developing a typology of interventions to support doctors’ mental health and wellbeing. BMC Health Serv Res. 2024, May, 3;24(1):573. 38702774 10.1186/s12913-024-10884-6 PMC11067176

