# Supplementary material for: Menopause and the healthcare workforce: a scoping review and stakeholder consultation
Source: BMC Health Serv Res. 2026 Jan 29;26:286. doi: 10.1186/s12913-025-13906-z (PMC12924456; doi:10.1186/s12913-025-13906-z)
Supplement: Supplementary file 1 — Supplementary Material 1 [file 12913_2025_13906_MOESM1_ESM.docx]

**Additional File 1:** Definitions:

We used the terms “women” or “female” throughout the article as that is the language currently used in the majority of published research. We acknowledge that not all women are assigned female at birth and that trans-men and non-binary people may experience symptoms of menopause. The terminology used here is not intended to isolate, exclude or diminish any individuals experience nor to discriminate against any group. The experiences described may be shared by all people undergoing the menopause transition, but we acknowledge that experiences may be different.

Perimenopause: the stage where there are changes in the menstrual cycle often associated with physical and psychological symptoms due to fluctuating and declining levels of oestrogen. Average age of perimenopause 45-55 years.

Menopause: the point at which there has been 12 months since a women’s last menstrual period. Average age of menopause is 51 years.

Post menopause: the stage which begins 12 months after a women’s last menstrual period.

Figure 4: graphic to illustrate the relationship between the different stages of the menopause

**
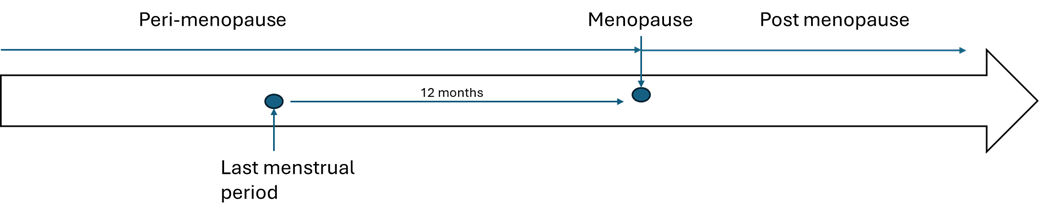
**
