# Supplementary material for: Menopause and the healthcare workforce: a scoping review and stakeholder consultation
Source: BMC Health Serv Res. 2026 Jan 29;26:286. doi: 10.1186/s12913-025-13906-z (PMC12924456; doi:10.1186/s12913-025-13906-z)
Supplement: Supplementary file 2 — Supplementary Material 2 [file 12913_2025_13906_MOESM2_ESM.docx]

**Additional File 2:**

**Health Personnel MeSH tree:**

Allied Health Personnel

Animal technicians
Athletic trainers
Community health workers
Dental Auxiliaries

Dental Assistants
 Dental Hygienists
 Dental Technicians
 Denturists

Anatomists 
Anesthetists +

Anesthesiologists
Nurse Anesthetists

Audiologists 
Caregivers 
Case Managers 
Coroners and Medical Examiners 
Dental Staff +

Dental Auxiliaries

Dental Assistants
 Dental Hygienists
 Dental Technicians
 Denturists

Dental staff, hospital
Dentists +

Dentists, Women
Endodontists
Oral and Maxillofacial Surgeons
Orthodontists

Doulas 
Epidemiologists 
Faculty, Dental 
Faculty, Medical  
Faculty, Nursing 
Health Facility Administrators

Hospital administrators

Chief Executive Officers, Hospitals

Infection Control Practitioners  
Medical Chaperones 
Medical Laboratory Personnel 
Medical Staff +

Hospitalists
Nurses +

Nurse administrator
Nurse Practitioners

Family nurse practitioners
Paediatric nurse practitioners

Nurse specialists

Nurse anesthestists
 Nurse clinicians
 Nurse midwives
 Nurses pediatric

Nurses, neonatal

Nurses, Community Health
 Nurses, International
 Nurses, Male
 Nurses, Public Health

Nursing staff

Nursing staff, Hospital

Nurtitionists 
Occupational Therapists  
Optomoetrists  
Hospital, Personnel

Dental Staff, Hospital
Hospital administrators

Chief Executive officers, Hospital

Hospital Volunteers
Medical Staff, Hospital

Hospitalists

Nursing staff, Hospital

Pharmacists 
Physical Therapists 
Physican Executives  
Physicians +

Allergists
Anesthesiologists
Cardiologists
Dermatologists
Endocrinologists
Foreign Medical Graduates
Gastroenterologists
General Practitioners
Geriatricians
Hospitalists
Nephrologists
Neurologists
Obstetricians
Occupational Health Physicians
Oncologists

Radiation Oncologists

Ophthalmologists
Osteopathic Physicians
Otolaryngologists
Pathologists
Paediatricians

Neonatologists

Physiatrists
Physicians, Family
Physicins, Primary Care
Physicians, Women
Psychiatrists
Pulmonologists
Radiologists

Radiation Oncologists

Rheumatologists
Surgeons

Barder Surgeons
Neurosurgeons
Oral and Maxillofacial Surgeons
Orthopedic Surgeons

Urologists

Psychologists
Psychotherapists
Traitionsl Medicine Practitioners 
Veterinarians

Search strategy **r**un on 20/11/2023.

**Ovid MEDLINE(R)**

| **#** | **Query** |
| --- | --- |
| **1** | **exp Menopause/** |
| **2** | **menopause.ab,ti.** |
| **3** | **1 or 2** |
| **4** | **exp Health Personnel/** |
| **5** | **health personnel.ab,ti.** |
| **6** | **4 or 5** |
| **7** | **exp Workplace/** |
| **8** | **workplace.ab,ti.** |
| **9** | **7 or 8** |
| **10** | **6 or 9** |
| **11** | **3 and 10** |

**Embase**

| **#** | **Query** |
| --- | --- |
| **1** | **exp menopause/** |
| **2** | **menopause.ab,ti.** |
| **3** | **1 or 2** |
| **4** | **exp health workforce/** |
| **5** | **exp hospital personnel/** |
| **6** | **exp paramedical personnel/** |
| **7** | **health personnel.ab,ti.** |
| **8** | **4 or 5 or 6 or 7** |
| **9** | **exp workplace/** |
| **10** | **workplace.ab,ti.** |
| **11** | **9 or 10** |
| **12** | **8 or 11** |
| **13** | **3 and 12** |

**HMIC Health Management Information Consortium**

| **#** | **Query** |
| --- | --- |
| **1** | **menopause.ab,sh,ti.** |
| **2** | **health personnel.ab,sh,ti.** |
| **3** | **workplace.ab,sh,ti.** |
| **4** | **2 or 3** |
| **5** | **1 and 4** |

**APA PsycInfo**

| **#** | **Query** |
| --- | --- |
| **1** | **menopause.ab,sh,ti.** |
| **2** | **health personnel.ab,sh,ti.** |
| **3** | **workplace.ab,sh,ti.** |
| **4** | **2 or 3** |
| **5** | **1 and 4** |

**Social Policy and Practice**

| **#** | **Query** |
| --- | --- |
| **1** | **menopause.ab,hw,ti.** |
| **2** | **health personnel.ab,hw,ti.** |
| **3** | **workplace.ab,hw,ti.** |
| **4** | **2 or 3** |
| **5** | **1 and 4** |

**CINAHL via Embase Host**

| **#** | **Query** |
| --- | --- |
| **S5** | **(S1 AND S4)** |
| **S4** | **(S2 OR S3)** |
| **S3** | **MW workplace OR TI workplace OR AB workplace** |
| **S2** | **MW health personnel OR TI health personnel OR AB health personnel** |
| **S1** | **MW menopause OR TI menopause OR AB menopause** |
