# Supplementary material for: Menopause and the healthcare workforce: a scoping review and stakeholder consultation
Source: BMC Health Serv Res. 2026 Jan 29;26:286. doi: 10.1186/s12913-025-13906-z (PMC12924456; doi:10.1186/s12913-025-13906-z)
Supplement: Supplementary file 3 — Supplementary Material 3 [file 12913_2025_13906_MOESM3_ESM.docx]

**Additional File 3:** Summary of all included articles

| **Author** | **Year** | **Empirical/**  **non-empirical** | **Study design** | **Country** | **Setting** | **Healthcare professional** | **N** | **Topic/ Aim of study** | **Main findings** |
| --- | --- | --- | --- | --- | --- | --- | --- | --- | --- |
| Adelekan-Kamara | 2023 | Empirical | Qualitative | UK | All healthcare | Doctors | 41 | To explore the interplay between factors underpinning an improved menopausal experience in the UK clinical workplace | Four meta-themes as the factors underpinning an improved menopause experience: Menopausal knowledge and awareness; Openness to discussion; Organisational culture; Supported personal autonomy.  Improved menopause experience was defined by participants as where individuals felt empowered to tackle the physical and cognitive challenges of menopause at work”. |
| Albuquerque | 2019 | Empirical | Cross-sectional (descriptive and analytic) | Brazil | Primary care | Nurses | 98 | To evaluate the quality of life of nurses who are experiencing the climacteric period and work in primary healthcare of a capital in the northeast region of Brazil. | Younger age group was associated with better quality of life across four domains.  Age 50-59 had worst quality of life levels with statistical significance in psychological, environmental and general domains. |
| Allkins | 2023 | Non empirical | Editorial | UK | - | Midwives | - | Supporting maternity workers with menopause - a recent publication from the Royal college of Midwives. | - |
| Anon  Nurse educator | 2009 | Non empirical | News | USA | - | Nurses | - | Good News for Nursing Faculty - a study that has shown that when in post menopause phase the cognitive function of women returns. | - |
| Anon  Nursing standard | 2014 | Non empirical | News | UK | - | Nurses | - | Let's talk about menopause at work - Royal College of Nursing vote to raise awareness of the effects of menopause in the nursing workforce. | - |
| Anon  British Dental Journal | 2021 | Non empirical | News | UK | - | Dentists | - | Campaign launched for menopause-friendly dental workplaces | - |
| Anon  Nursing management | 2021 | Non empirical | Comment | UK | - | Nurses | - | Support for menopause symptoms at work. | - |
| Anon  Nursing standard | 2022 | Non empirical | Briefing | UK | - | Nurses | - | Get workplace support during menopause | - |
| Anon  Frontline | 2024 | Non empirical | News | UK | - | Nurses | - | Menopause in the workplace. Menopause isn’t a protected characteristic legally requiring workplace adaptations. However, with support of workplace safety reps nurses can be supported to seek help through occupational health. | - |
| Banks | 2019 | Non empirical | Review | UK | Acute care trust | Mixed | - | Menopause and the NHS: caring for and retaining the older workforce, how a trust has implemented interventions to support staff in menopause. | - |
| Bapayeva | 2023 | Empirical | Cross-sectional (descriptive) | Kazakhstan | University hospitals | Doctors Nurses | 130 | To investigate the relationship between the work environment and the menopausal quality of life in physicians and nurses | Doctors with managerial positions were significantly more bothered by vasomotor symptoms and had higher psychological scores.  Satisfaction with work environment was associated with better quality of life related to psychological symptoms in nurses. Nurses who were satisfied with their salary were less bothered by physical symptoms. |
| Barker | 2024 | Non empirical | Clinical discussion | UK | - | Dentists | - | Managing Menopause, a review of menopause and symptoms with discussion of how dental nurses can support their colleagues. | - |
| Bates | 2021 | Non empirical | Comment | UK | - | Nurses | - | Discussion of the taboo around menopause and asking for the NHS to be more considerate. | - |
| Beesley | 2022 | Non empirical | News | UK | - | Midwives | - | NHS employers are failing staff on menopause support says the Royal College of Midwives calling for urgent improvements | - |
| Bell | 2022 | Non empirical | Opinion | UK | - | Dentists | - | Menopause is an issue in dentistry but there is no literature on the effects that menopause can have on the dental team. | - |
| Benetti | 2019 | Empirical | Qualitative | Brazil | Family health unit | Mixed | 15 | To describe how the women investigated perceived climacteric symptoms in their place of work as well as to analyse the strategies used to manage and cope with this stage of life. | Menopause is different for different people with regards to symptoms but also meanings and how they feel about it.  The menopause can cause interpersonal difficulties with colleagues and have an impact on their ability to work. People have different coping strategies including exercise, food, religiosity and medical care. |
| Beneventi | 2021 | Empirical | Cross sectional (descriptive) | Brazil | All healthcare | Nurses | 218 | To identify the morbidities and medications used by nurses practicing in Sao Paulo during the climacteric period to raise awareness and contribute to future preventive health measures in this population | The most common symptoms of menopause were physical and mental exhaustion and muscle and joint issues. Results can support development of preventative measures aimed at the main risk factors for morbidities to which nurses are exposed to during the menopause. |
| Blumel | 1995 | Empirical | Cohort | Chile | Acute care hospital | Mixed | 444 | To compare the average number of days absent from work in climacteric women with and without HRT. | Post menopausal women who take oestrogen regularly have half the number of days of absence than those who do not |
| BMA | 2020 | Non empirical | Report | UK | - | Doctors | - | Challenging the culture on menopause for working doctors British Medical Association report | - |
| Calow | 2023 | Empirical | Literature review | Mixed | All healthcare | Nurses | - | To undertake a review of the literature to determine the level of knowledge about menopause that healthcare students are exposed to and highlight why it is important for them to understand this subject for their own clinical practice and supporting colleagues in the workplace | The extent of symptoms and levels of discrimination is forcing women out of the workforce. Workplace guidance exists but it is unlikely that women are receiving adequate workplace support. |
| Cao | 2014 | Empirical | Cross sectional (descriptive) | China | Hospital | Nurses | 540 | To study the occurrence and influencing factors of perimenopausal syndrome in clinical nurses in tertiary hospitals in Tangshan City. | 63% of nurses report perimenopausal syndrome.  Nurses with a high work pressure and intensity tended to have more severe perimenopausal syndrome. |
| Cavalcante | 2006 | Empirical | Qualitative | Brazil | Diabetes unit | Mixed | 26 | To analyse the interference of problems experienced by women during the climacteric periods and their relationship with health and the work environment. | 19 of 26 participants expressed knowledge of the physiological changes and symptoms of menopause Four sought out medical advice due to symptoms of menopause. 19 stated that the symptoms interfere with work and family relationships with tiredness, irritability and hot flushes being the symptoms that most interfered with work. |
| Converso | 2019 | Empirical | Cross sectional (descriptive) | Italy | Public hospitals | Nurses | 94 | To examine the relationship between menopausal symptoms and job burnout in a sample of women during the menopause. | Menopausal symptoms were positively correlated with emotional exhaustion and depersonalization.  No social or social resources moderate the effect on emotional exhaustion. However, support from superiors and colleagues, optimism and resilience moderated the detrimental effects from menopausal symptoms on depersonalization. |
| Cornock | 2022 | Non empirical | Opinion | UK | - | Mixed | - | Menopause: how reasonable adjustments could help healthcare staff at work | - |
| Cornock | 2022 | Non empirical | Expert advice | UK | - | Mixed | - | Menopause symptoms: the adjustments at work that could help | - |
| Critchley | 2021 | Non empirical | Review | UK | - | Doctors | - | The female medical workforce, description of the issues effecting female doctors | - |
| Cronin | 2022 | Empirical | Qualitative | Mixed | All healthcare | Nurses | 48 | To explore and discuss the suitability of digital health interventions as strategies to facilitate coping and alleviate discomfort in menopausal women.  Examine how digital health strategies could be incorporated as support and health interventions to support women in the nursing workplace. | Four themes were identified: managing symptoms in the workplace, recognition in the workplace, menopause interventions, and expectation versus the invisible reality. Four themes were felt to be important in terms of the intervention: connection, information, tracking, evidence-based information on interventions for specific symptoms. |
| Cronin | 2023 | Empirical | Qualitative | UK | Hospital trust | Mixed | 174 | To explore and understand the organizational culture of a workplace in terms of support and well-being for staff experiencing perimenopausal and menopausal symptoms at work | High numbers of staff experienced symptoms of menopause at work. There was variability among the staff around how supportive they found their managers and how available support and adjustments were.  Symptoms severity increased with work related stress.  Employees felt uncomfortable discussing it with managers, but managers felt they were approachable.  Managers felt that they would respond to discussions about symptoms, but employees didn’t report having the discussions.  Two main themes came out of interviews: access to support and culture of menopause |
| Davies | 2022 | Non empirical | Guidelines/ Consensus document | UK | - | Doctors | - | Age and the anaesthetist: considerations for the individual anaesthetist and workforce planning | - |
| Dean | 2018 | Non empirical | Features | UK | - | Nurses Midwives | - | Menopause: why nurses need help from their employers | Employers have been slow to recognise the need to support nurses in menopause. |
| Dean | 2019 | Non empirical | Analysis | UK | - | Nurses | - | Calling time on a culture of silence, women with menopause symptoms at work deserve support and empathy in the workplace | - |
| Dean | 2020 | Non empirical | Analysis | UK | - | Nurses | - | Menopause at work: managing hot flushes and PPE | - |
| Devlin | 2019 | Non empirical | Comment | UK | - | Nurses | - | Who's helping nursing through menopause? Health services risk losing experienced nurses because of failure to acknowledge symptoms such as fatigue and hot flushes. | - |
| Ding | 2022 | Empirical | Qualitative | China | Secondary and tertiary hospitals | Nurses | 16 | To provide evidence for exploring interventions to promote the physical and mental health of perimenopausal nurses. | Factors influencing health of menopausal nurses: Patient factors (serious conditions, lack of understanding) Working environment factors (inadequate staff, poor competency of new nurses, management, income distribution)  Individual factors (Inadequate self-empowerment and inability to do the job, being introvert, sensitive and suspicious)  Family and social factors. |
| dos Reis | 2011 | Empirical | Cross sectional (descriptive) | Brazil | Hospital | Mixed | 385 | To identify the signs and symptoms caused by the climacteric and its interference in the work process of professionals at a university hospital. | Main symptoms that nurses felt interfered with work were: pain in arms, back and legs, agitation, excessive tiredness, headache and reduced memory |
| Fonseca | 2014 | Empirical | Qualitative | Brazil | University hospitals | Nurses | 9 | Analyse the repercussions of climacteric in the quality of life of nursing professionals who act directly in assistance. | Nurses reported that climacteric symptoms interfered with work because of physical tiredness, alterations of humour or difficulties with the team.  It was felt that the long hours needed to earn enough money from nursing were not possible due to age and symptoms of menopause. |
| Giron | 2012 | Empirical | Qualitative | Brazil | University hospitals | Nurses | 9 | To identify the physical effects of the climacteric on professional nurses who are involved directly in healthcare | Exhausting working hours associated with the physical effects of the menopause increased the chances of alterations in nurses' personal and professional lives.  Hot flushes and mood swings were symptoms that most annoyed the women. |
| Hamoda | 2021 | Non empirical | Letter to editor | UK | - | Doctors | - | BMS, RCOG, RCGP, FSRH, FOM and FPH Position Statement in response to the BMA report ‘Challenging the culture on menopause for doctors' | - |
| Hickey | 2017 | Empirical | Cross Sectional (descriptive) | Australia | Large hospitals | Mixed | 1092 | To determine the relationship between menopausal symptoms and work outcomes | The three most common symptoms were sleep, joint and muscular pain, physical and mental exhaustion.  No significant differences between women at different menstrual stages in relation to work engagement, affective commitment, satisfaction, limitations and perceived supervisor support.  Temperature control, flexible working hours and seminars about healthy aging were the most commonly chosen suggestion for support. |
| Hill | 2020 | Non empirical | News | UK | - | Doctors | - | Female doctors in menopause retiring early due to sexism, says study (BMA report) | - |
| Hobson | 2024 | Empirical | Qualitative | UK | All healthcare | Mixed | 14 | To explore the impact of the menopause on the working lives of NHS staff working in Wales with specific emphasis on their experience of menopausal symptoms and management strategies in the workplace. | Three themes were identified: experiences of menopausal symptoms and symptom management; impact of menopause on work; and impact of work on the menopause |
| Holland | 2022 | Non empirical | Feature | UK | - | Dentists | - | Taking the taboo out of the menopause - how dentistry is grappling with "the change". | - |
| Kitney | 2023 | Non empirical | Guidance and advice | UK | - | Mixed | - | Being a menopause friendly employer, advice from the Dental Defence Union. | - |
| Kydd | 2021 | Non empirical | Editorial | UK | - | Mixed | - | Menopause and personal protective equipment: How does this meet acceptable working conditions? | - |
| Lei | 2015 | Empirical | Cross sectional (descriptive) | China | Tertiary Hospital | Nurses | 429 | To mark the impact of work environment and social support on perimenopausal syndrome in clinical nurses and promote this exhibition for clinical nursing intervention. | Stronger light and greater noise decibel was associated with frequency and severity of moderate to severe perimenopausal syndrome |
| Matsuzaki | 2014 | Empirical | Cross sectional (descriptive) | Japan | Public and private hospitals | Nurses | 1169 | To investigate menopause symptoms and job-related stress in Japanese registered nurses at the menopausal transition.  To investigate the differences in menopausal symptoms and job-related stress among nurses in managerial positions and those not in managerial positions | Menopausal symptoms were associated with job related stress. There were differences between the factors that lead to job related stress between nurses in managerial positions and those not. |
| Matsuzaki | 2016 | Empirical | Cross Sectional (descriptive) | Japan | Public and private hospitals | Nurses | 732 | To examine the differences in coping with menopausal symptoms between nurses and general workers.  To examine the associations of the difference with understanding menopausal symptoms in Japan. | The proportions of nurses and general workers coping with menopausal symptoms were similar (around 50%). Nurses had a better knowledge of the menopause. A variety of coping strategies were used, none of which were interventions provided by the workplace. |
| Matsuzaki | 2019 | Empirical | Cross Sectional (descriptive) | Japan | Public and private hospitals | Nurses | 1174 | To examine the associations of personality with menopausal symptoms, use of HRT and coping with menopausal symptoms in Japanese nurses. | There were differences in the menopausal symptoms and coping strategies between women with type A personality and non-type A personality. The proportion of nurses receiving HRT with type A personality was higher than with non-type A. There were no significant differences between the two groups with other coping strategies. |
| Membrive | 2011 | Empirical | Cross Sectional (descriptive) | Spain | Primary care and hospitals | Nurses | 86 nurses 117 teachers | To describe and analyse the association between labour conditions and quality of life among climacteric women working in health and education. | Working in healthcare in the perimenopause is associated with lower quality of life than working in education. Nurses who were most satisfied with their work displayed a better quality of life. Stress and exposure to noise determined a worse quality of life. Exposure to a comfortable temperature in the workplace was related to an increase in quality of life. |
| Morris | 2023 | Non empirical | News | UK | - | Mixed | - | Scottish government launches new women's health policy across NHS Scotland | - |
| MPS | 2020 | Non empirical | Policy paper | UK | - | Doctors | - | Supporting doctors through the menopause | - |
| Noble | 2019 | Non empirical | Feature | UK | - | Nurses | - | Making the menopause more manageable, a nurse led scheme offering employees in Wales specialist clinics | - |
| Noble | 2021 | Non empirical | Review | UK | - | Nurses | - | Helping and supporting staff to manage menopause symptoms at work | - |
| Norton | 2019 | Non empirical | Review | UK | - | Nurses | - | How nurse leaders can support staff going through the menopause | - |
| Nowakowska | 2021 | Empirical | Cross sectional (descriptive) | Poland | Hospital | Nurses | 169 | To present the opinions of professionally active perimenopausal nurses on the assessment of the quality of working life from the point of view of selected factors of work environment and their self-efficacy. | No relationship observed between work organization and the assessment of self-efficacy.  Most of the women rated self-efficacy highly despite various assessments of organisational factors in the hospital. |
| O'Hearn | 2022 | Empirical | Qualitative case study | Canada | Mixed settings | Physiotherapists | 29 | To increase the understanding of the gendered dimensions of health and wellbeing concerning the transition to menopause within the work environment. How to place-based experiences in the work environment impact physiotherapists undergoing the transition to menopause? | Three main themes: - Being well at work - finding a balance, transformative experience, redefining their purpose - The embodied experience - unpredictable body, exploring the invisible, normalizing the transition. - Navigating supports - reflective practice, community of support, institutional structures. |
| O'Neill | 2023 | Empirical | Cross sectional (descriptive) | Ireland | Hospital | Mixed | 339 | To measure the prevalence of menopausal symptoms in employees in a healthcare setting To assess the impact of individual symptoms on work, attendance and career development.  To explore perceptions about workplace supports | Female employees are negatively impacted by menopausal symptoms at work.  The most common symptoms affecting employees were fatigue, difficulty sleeping, poor concentration, and poor memory.  65% stated that symptoms had affected their work performance, 35% reported career development decisions were influenced and 18% had taken sick leave.  Manager awareness was the most important workplace support identified, followed by flexible working times. |
| O'Sullivan | 2023 | Non empirical | Letter | UK | - | Doctors | - | Pause for thought: the impact of the menopause on women in the workplace, focus on surgeons. | - |
| Pan | 2014 | Empirical | Cross sectional (descriptive) | China | Hospital | Nurses | 107 | To discuss the menopausal symptoms' impact factors of clinical nurses in Tangshan city and provide a reference for better health guidance to improve the quality of life and work in menopausal nurses. | Nurses with high work pressure, low job satisfaction, a history of chronic disease, introversion, poor sleep quality and low life satisfaction had higher incidence of menopause symptoms. |
| Pitman | 2022 | Empirical | Cross sectional (descriptive) | Ireland | All healthcare | Nurses Midwives | 1045 | To gain an understanding of nurses and midwives' experience of menopause in the workplace. | Menopause is an important occupational issue.  Menopause symptoms are impacting nurses at work. 17% indicated they had missed work with 43% reporting they have missed approx. 5 days due to symptoms and 82% have considered leaving work or reducing their hours. |
| Prothero | 2021 | Empirical | Mixed methods | UK | Ambulance trust | Ambulance service | 522 | Explore the impact of the menopause transition on women working within one UK ambulance trust, to inform the need of menopause related well-being support. | The impact of menopause on ambulance staff was significant. The most challenging symptoms identified were tiredness/ low energy levels, difficulty sleeping, and mood changes.  Respondents had needed time off but only half informed their manager of the real reason for absence.  Awareness is lacking and there is clear scope for improved support and initiatives. |
| Riach | 2023 | Empirical | Mixed methods | UK | All healthcare | Mixed | 6453 | 1. Capture the experience of menstrual and menopausal health at work for NHS Scotland employees 2. Understand the needs of women and people who menstruate, surrounding menstrual health and menopausal health support at work 3. Generate evidence-based recommendations around best practice based on employees lived experiences | Instances of misdiagnosis, underdiagnosis and late diagnosis remained a challenge and had an influence of women's careers and capacity to work. Employees were creative and resilient in how to work through pain and were often able to manage disruption due to menstrual or menopausal experiences through "micro-accommodations", particularly when supported by colleagues and line managers.  Organisational culture played a significant role in employees experiences of reproductive health at work. |
| Riach | 2021 | Empirical | Qualitative | Australia | All healthcare | Mixed | 1092 | To use an intersectional lens to explore menopausal experience of women working in the higher education and healthcare sectors in Australia. | Menopausal support at work can slip into "managing" menopause which risks consigning menopause to HR and other short term initiated that commoditize women's bodies while not transforming cultures. |
| Rodrigues | 2020 | Empirical | Qualitative | Brazil | Hospital | Mixed | 12 | To develop a better understanding of the difficulties of professional women during the climacteric period | Two themes: 1. Hot flashes as a risk factor for depression 2. Unwellness or lack of sexual desire as an expression of low self esteem  Not everyone presents in the same way.  Climacteric syndrome negatively influences personal relationships at work. |
| Schott-Baer | 2000 | Empirical | Cross sectional (descriptive) | USA | Hospital | Mixed | 462 | To evaluate the 12 most common actions recommended to reduce menopause symptoms | Environmental self-care actions can reduce symptoms experienced by women not taking HRT. |
| Stock | 2019 | Empirical | Cohort | USA | All healthcare | Nurses | 80840 | To investigate the association between rotating night shift work and age at natural menopause within the Nurses Health Study 2 cohort. | Working rotating night schedules was less common with age Moderately increased risk of earlier menopause for women with >10 months of rotating night shift work in the previous 2 year period. Working 11-20 years conveyed a slightly higher risk of earlier menopause compared to having never worked night shifts. |
| The strategy unit | 2022 | Empirical | Mixed methods | UK | NHS organisations | Mixed | - | What does the menopause cost the NHS as an employer? | Menopausal related symptoms can affect how individuals participate in the NHS workforce.  Managing menopausal symptoms at work is difficult.  There is a significant financial cost associated with the menopause for the NHS as an employer. The annual cost of menopausal symptoms may be between £89-£129 million. |
| The strategy unit | 2023 | Empirical | Qualitative | UK | NHS organisations | Mixed | 76 | To understand what it is like to experience the menopause as a NHS employee | Menopausal symptoms impact participants ability to work.  Commonly reported symptoms included brain fog or memory loss. Confidence is impacted with some participants stating they no longer felt they could carry out their roles to the same perceived competency. Menopause-related sickness absence, presenteeism and leaving the workforce is highly reported. |
| The strategy unit | 2023 | Empirical | Cross sectional (descriptive) | UK | NHS organisations | Mixed | - | What are the characteristics of the NHS women of menopausal age | 1604 days (out of 4.4million) of sickness were recorded related to menopause with 77% of these days being by a women aged 45-55.  Mental health is primary reason in the majority of the days. There is very low use of the menopause recording for sickness (available for 2 years at time of publication). |
| Thomas | 2022 | Non empirical | Advice | UK | - | Nurses | - | Advice nurse who has been through the menopause. | - |
| Vallejo | 2016 | Empirical | Cross sectional (descriptive) | Mixed | Hospital | Doctors | 947 | To evaluate whether menopausal status and symptoms among female gynaecologists would influence their clinical behaviour related to menopausal hormone therapy (MHT) | Most female gynaecologists in the survey would use menopausal hormone therapy (MHT) if menopause symptoms were present.  Post menopausal women prescribe MHT to their symptomatic patients at a higher rate than premenopausal women. |
| Vanderzalm | 2023 | Empirical | Qualitative | Canada | Hospital | Nurses | 13 | Examine acute care nurses' experience of menopause and perimenopause in relation to their caregiving abilities. | Three themes: nursing during this stage of life; navigating life at work; workplace adaptations. |
| Waters | 2022 | Non empirical | News | UK | - | Mixed | - | NHS must offer flexible working to staff with menopausal symptoms says guidance | - |
| Webber | 2022 | Non empirical | News | UK | - | Mixed | - | NHS menopause guidance recommends flexible working | - |
| Wood | 2021 | Non empirical | Advice | UK | - | Care home workers | - | Tips of hormone health for care workers | - |
